# Examining lung mechanical strains as influenced by breathing volumes and rates using experimental digital image correlation

**DOI:** 10.1186/s12931-022-01999-7

**Published:** 2022-04-11

**Authors:** C. A. Mariano, S. Sattari, K. A. M. Quiros, T. M. Nelson, M. Eskandari

**Affiliations:** 1 Department of Mechanical Engineering, University of California at Riverside, Riverside, CA USA; 2 BREATHE Center, School of Medicine, University of California at Riverside, Riverside, CA USA; 3 Department of Bioengineering, University of California at Riverside, Riverside, CA USA

**Keywords:** Digital image correlation, Topological strain, Pulmonary mechanics, Anisotropy, Heterogeneity, Biomechanics, Ventilation, Inflation

## Abstract

**Background:**

Mechanical ventilation is often employed to facilitate breathing in patients suffering from respiratory illnesses and disabilities. Despite the benefits, there are risks associated with ventilator-induced lung injuries and death, driving investigations for alternative ventilation techniques to improve mechanical ventilation, such as multi-oscillatory and high-frequency ventilation; however, few studies have evaluated fundamental lung mechanical local deformations under variable loading.

**Methods:**

Porcine whole lung samples were analyzed using a novel application of digital image correlation interfaced with an electromechanical ventilation system to associate the local behavior to the global volume and pressure loading in response to various inflation volumes and breathing rates. Strains, anisotropy, tissue compliance, and the evolutionary response of the inflating lung were analyzed.

**Results:**

Experiments demonstrated a direct and near one-to-one linear relationship between applied lung volumes and resulting local mean strain, and a nonlinear relationship between lung pressures and strains. As the applied air delivery volume was doubled, the tissue surface mean strains approximately increased from 20 to 40%, and average maximum strains measured 70–110%. The tissue strain anisotropic ratio ranged from 0.81 to 0.86 and decreased with greater inflation volumes. Local tissue compliance during the inflation cycle, associating evolutionary strains in response to inflation pressures, was also quantified.

**Conclusion:**

Ventilation frequencies were not found to influence the local stretch response. Strain measures significantly increased and the anisotropic ratio decreased between the smallest and greatest tidal volumes. Tissue compliance did not exhibit a unifying trend. The insights provided by the real-time continuous measures, and the kinetics to kinematics pulmonary linkage established by this study offers valuable characterizations for computational models and establishes a framework for future studies to compare healthy and diseased lung mechanics to further consider alternatives for effective ventilation strategies.

## Background

The global pandemic has pushed pulmonary mechanics research to the forefront. Prior to COVID-19, respiratory illnesses were already one of the leading causes of death worldwide [1, 2]. Burdensome respiratory ailments such as chronic obstructive pulmonary disease (COPD) are the leading causes of disability amongst an expansive list of lung diseases which includes tuberculosis and acute lower respiratory tract infection, all detrimental to lung function [3]. Other examples of respiratory illnesses affecting countless individuals which lead to irreversible tissue damage are pneumonia, asthma, and emphysema [4–6].

Severe manifestations of such respiratory diseases can often lead to the need for additional breathing support in the form of mechanical ventilation (MV). Preexisting lung conditions play a major role in inflicting tissue damage when mechanically ventilated, and the risk of ventilator induced lung injuries (VILI), such as barotrauma and edema, are greater [7]. Another possibility of MV is ventilator-associated pneumonia which affects 8–28% of patients with high chances of obtaining this complication after just 48 h on ventilation, demonstrative of the quick onset of complications due to ventilation [8, 9].

Factors that contribute to these injuries are heavily related to the ventilator driving pressure as well as the mechanical elasticity, integrity, and behavior of the lung tissue when ventilated, which is compromised in diseased states [10–13]. The behavioral mechanics of lung tissue has been explored through investigations of isolated parenchymal strips and tracheal and bronchial airway segments via tensile [14–16], biaxial [17, 18], and indentation tests [19–21]. However, there are limited studies considering the local stretch behavior of tissue in situ, where the tissue still resides on the intact lung organ. The local tissue behavior in response to globally applied variable inflation volumes and breathing rates is particularly key to understanding strain heterogeneities and concentrations [22, 23], and can support studies focusing on alternative ventilation strategies to minimize VILI (e.g. multi-oscillatory and high-frequency ventilation) [24, 25].

As such, the objective of this current study is to characterize ventilation strains. We do so by utilizing digital image correlation (DIC) as a novel technique for analyzing topological deformations of the inflating whole porcine lung organ. Building upon previously introduced methods, we interface two systems to simultaneously assess local strain and global pulmonary measurements throughout the lung expansion cycle [26, 27]. Existing strain measurement studies, such as digital volume correlation (DVC), optical coherence tomography (OCT) [28–30], computerized tomography (CT) scans [31, 32] and doppler elastography and speckle tracking for biological tissues [33, 34] are limited because they are at discrete time points, retrieve 2D contour images with projected strains, or are time intensive, which affects the resulting measured lung behavior.

In contrast, in this study we employ DIC as a real-time continuous imaging approach to examine lung deformations instantaneously and divulge regional pulmonary behaviors, such as tissue strain heterogeneity and isotropy, and further investigate volume and rate ventilation effects on these lung behaviors. The resulting comprehensive characterization of local–global lung mechanics in pig specimens offers critical pulmonary insights for new oscillatory ventilation schemes. Given the similarity between porcine and human lung size and anatomy, findings can help support studies aimed at lowering the risk of ventilation injuries and complications [35]. Furthermore, the variable inflation volume and breathing rate data collected in this work can inform finite element computational models to enable predictive ventilation techniques [36].

## Methods

### Sample preparation

Pulmonary samples were obtained from four pigs (A, B, C, and D) weighing approximately 200–250 lbs, ranging 6–8 months in age (local abattoir, IACUC approval not required). These specimens were shipped with wet ice and stored in a plastic container, enveloped in a barrier of bubble wrap to protect the tissue from direct contact with the ice. Testing was conducted immediately upon arrival and within 36 h postmortem.

**Fig. 1:**
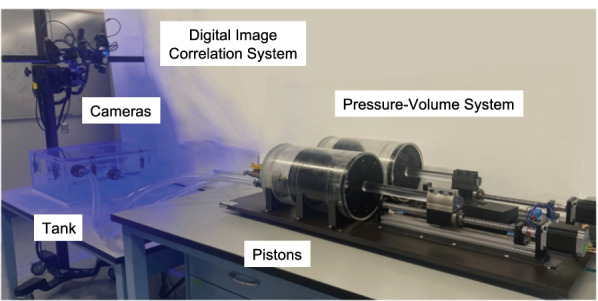
Experimental set-up of the electromechanical pressure–volume ventilation apparatus (right) interfaced with the digital image correlation system (left). The Trilion ARAMIS Adjustable 12M two-camera system is positioned above a transparent, air-tight tank containing the lung specimen which is controlled by the dual-piston apparatus to apply inflation volumes and measure resulting lung volumes and pressures. The combination of these two systems enables the simultaneous collection of global pressures and volumes and local lung topological strain measurements

Each lung specimen was weighed, and the initial lung volume was measured based on liquid submersion [37]. The lung was inflated by a laboratory air-line pressure outlet (< 1 psi) to open collapsed airways and to expand the surface for the speckling process and digital image correlation (DIC) technique previously detailed in Mariano et al. (2020) [27]. Briefly, a thin layer of quick-drying white enamel paint (rust-oleum) and an exfoliator pad was used to apply a randomized speckling pattern onto the specimen surface. The speckled lung was then placed inside our custom electromechanical pressure–volume (PV) system within an air-tight transparent tank for imaging and DIC data collection (Trilion ARAMIS Adjustable 12M system) as seen in Fig. 1 [26].

The volume of trapped air within each lung was calculated by subtracting the initial lung volume from its total volume computed by using the weight density equation, where the lung tissue density of 1.06 g/ml was used [38]. The peak lung volume and pressure were measured at the highest applied inflation volume’s (1350 ml) maximum values (Table 1).

**Table 1:** Specimen characteristics for varying breathing rates

Pig specimen	Weight [g]	Initial lung volume [ml]	Air trapped [ml]	Rate [breaths per minute]	Peak lung volume for 1350 ml applied volume [ml]	Peak pressure for 1350 ml applied volume [psi]
A	789.40	1620	875.28	10	–	–
				15	1215.34	0.40
				20	1197.10	0.45
B	818.96	1485	712.40	10	1215.34	0.36
				15	1199.38	0.42
				20	1185.70	0.46
C	1217.86	2160	1011.08	10	1165.17	0.37
				15	1121.85	0.38
				20	1064.85	0.42
D	783.96	1080	340.42	10	1126.41	0.47
				15	1105.89	0.42
				20	1023.80	0.52
Average	902.55 ± 210.77	1586.25 ± 446.05	734.80 ± 289.89	10	1168.97 ± 44.59	0.4 ± 0.06
				15	1160.62 ± 54.78	0.41 ± 0.02
				20	1117.86 ± 86.68	0.46 ± 0.04

Characteristics, and peak lung volume and pressures at maximum inflation volume at varying breathing rates of 10, 15, and 20 breaths per minute.   Lung volume was found to decrease while pressures were found to increase with faster breathing rates

### Experimental protocol and data collection

The sample was hydrated with 1XPBS solution atop a frictionless platform. Using our custom-built PV system depicted in Fig. 1 (design described in detail in Sattari et al. 2020), a preload of 0.05psi ensured common initial states between lung specimens [26]. The specimens underwent three preconditioning cycles inflated to specified volumes before the fourth cycle was analyzed [39]. The sample was inflated within the total lung capacity of porcine lung to three maximum volumes of 675, 900, and 1350 ml corresponding to tidal volumes of 6, 8, and 12 ml/kg in clinical practice and at rates of 10, 15, and 20 breaths per minute (BPM) [40, 41].

DIC data was collected at frequencies corresponding to the rate of inflation as follows: 10 Hz for 10 BPM, 15 Hz for 15 BPM, and 20 Hz for 20 BPM. The total dimensional DIC measuring volume was 375 × 295 × 295 mm. A facet size of 30 pixels and point distance of 10 pixels were used across all samples. Surface components were created at the maximum inflation stage of the lung to analyze the entire surface at full expansion as previously described [27]. The principal strains and stretches, major and minor, were calculated based on the reference stage of the uninflated, preconditioned state of the specimen for each volume and rate combination, which were then averaged across all lungs for each set of testing parameters.

### Data analysis

**Fig. 2:**
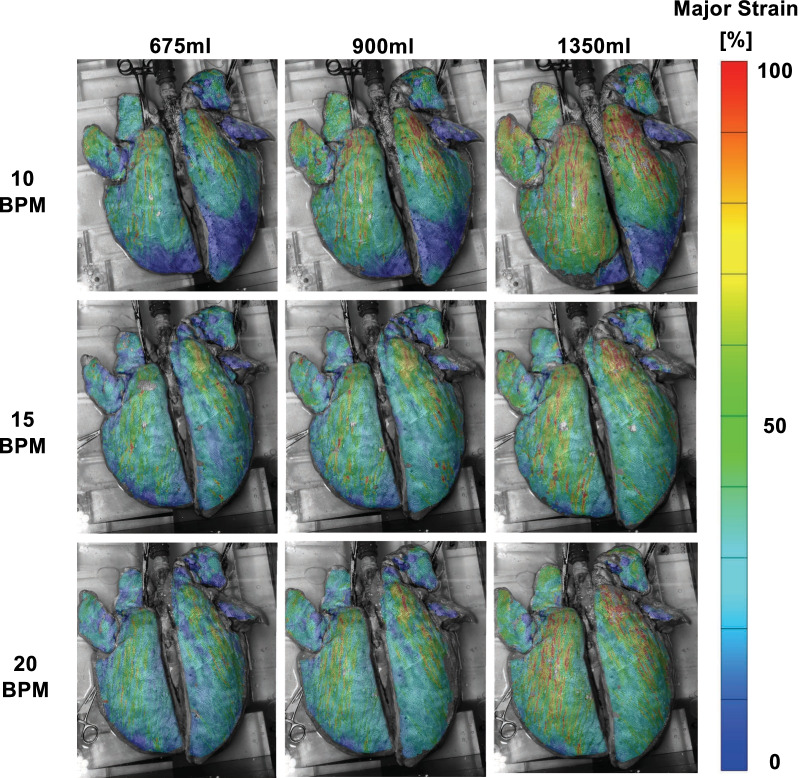
Topological strain contour maps of representative porcine lung at varying breathing rates of 10, 15, and 20 breaths per minute and inflation volumes of 675 ml, 900 ml, and 1350 ml at the maximum inflation stage. As the applied volume increased, surface strain heterogeneity became more prominent as indicated by appearance of regional high strain concentrations in the upper caudal left lung lobe and middle lobe of the right lung. The influence of breathing rate on local strains was not observed to be unidirectional

**Fig. 3:**
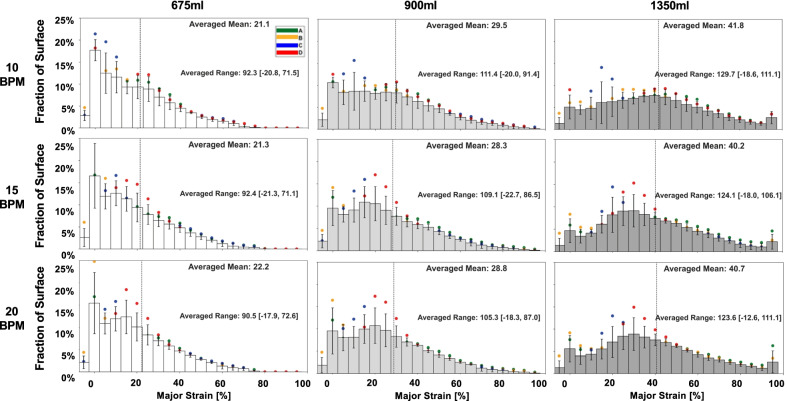
Averaged quantified strains across the fraction of the specimen’s topological surface at the maximum inflation stage divulged the effects of varying breathing rate and inflation volumes. Surface strains increased with increasing inflation volumes. Lower inflation volumes exhibited right skewed distribution and spread to a centered distribution at higher volumes, indicative of expanding strain ranges and heterogeneity. No statistical rate dependency was found, however the strains did depend on the applied volume (see Fig. 4)

Strain data collected from commercial GOM software (Trillion, King of Prussia, Pennsylvania, USA) was plotted as topological color contour maps to observe qualitative lung inflation patterns and visually assess the localized strains (Figs. 2, 7B). Histograms of the strain values at the maximum inflation stage were quantified for the various inflation volumes and multiple breathing rates to assess the heterogenous and isotropic tissue behaviors relative to their corresponding fraction of the lung surface (Figs. 3 and 5).

Table 2 documents individual specimen’s mean, median, maximum, and range of major principal strains at maximum inflation. The anisotropic ratio was calculated as the fraction of minor to major stretch which resulted in a ratio range of 0–1, where unity represents isotropic stretch. Outlying strain values were marked using the interquartile range analysis [42, 43].

**Table 2:** Individual and averaged specimen strain data measures and anisotropic ratio

Rate	Pig	Applied volume [ml]	Mean strain [%]	Median strain [%]	Max strain [%]	Strain range [%]	Averaged anisotropic ratio [–]	Rate	Pig	Applied Volume [ml]	Mean Strain [%]	Median Strain [%]	Max Strain [%]	Strain range [%]	Averaged anisotropic ratio [–]	Rate	Pig	Applied volume [ml]	Mean strain [%]	Median strain [%]	Max strain [%]	Strain range [%]	Averaged anisotropic ratio [–]
10BPM	A	675	22.2	20.8	70.8	88.7	0.84	15BPM	A	675	24.3	21.1	80.7	107.3	0.84	20BPM	A	675	23.7	20.5	78.7	100.4	0.84
		900	29.6	28.7	87.6	100.0	0.81			900	31.9	29.5	97.5	124.9	0.81			900	31.5	28.5	97.7	115.4	0.81
		1350	47.7	47.3	115.6	132.0	0.77			1350	46.3	44.9	118.8	143.1	0.78			1350	45.1	42.3	127.0	140.0	0.78
	B	675	20.0	16.6	67.6	83.1	0.87		B	675	15.8	10.1	63.1	81.3	0.89		B	675	19.7	15.0	75.6	93.9	0.87
		900	26.8	24.5	83.8	106.1	0.84			900	26.7	23.4	93.4	121.7	0.85			900	26.5	23.0	95.8	119.1	0.85
		1350	34.9	34.2	96.8	118.6	0.83			1350	38.4	37.6	116.8	134.0	0.82			1350	39.4	39.2	125.1	135.2	0.82
	C	675	18.4	11.6	69.9	96.4	0.87		C	675	22.5	16.2	79.3	103.7	0.87		C	675	21.9	16.2	75.4	94.5	0.87
		900	28.6	19.4	99.9	119.9	0.85			900	26.9	20.3	87.9	112.1	0.85			900	27.2	21.9	84.3	108.6	0.86
		1350	39.2	31.7	118.1	135.7	0.81			1350	39.2	33.7	106.8	131.1	0.82			1350	37.1	32.4	99.7	118.8	0.83
	D	675	24.0	23.2	77.6	101.0	0.85		D	675	22.7	20.9	61.4	77.4	0.85		D	675	23.5	21.3	60.6	73.3	0.85
		900	33.2	32.0	94.5	119.6	0.83			900	27.5	25.6	67.1	77.8	0.84			900	30.2	27.9	70.3	78.2	0.84
		1350	45.4	45.8	113.9	132.5	0.81			1350	36.8	34.7	82.0	88.4	0.82			1350	41.1	38.0	92.5	100.4	0.81
Average		675	21.1	18.1	71.5	92.3	0.86	Average		675	21.3	17.1	71.1	92.4	0.86	Average		675	22.2	18.3	72.6	90.5	0.86
		900	29.5	26.2	91.4	111.4	0.83			900	28.3	24.7	86.5	109.1	0.84			900	28.8	25.3	87.0	105.3	0.84
		1350	41.8	39.8	111.1	129.7	0.80			1350	40.2	37.7	106.1	124.1	0.81			1350	40.7	38.0	111.1	123.6	0.81

Mean, median, maximum, and range of strains, as well as anisotropic ratio, for each individual specimen under varying applied volumes of 675, 900, and 1350ml, organized by their corresponding breathing rate

Associations between local and global measurements of mean strain, applied volumes, and lung pressures were collected and analyzed throughout inflation. The local compliance, the relationship between local tissue strains and lung pressures was bilinear (Fig. 6). To calculate the initial (slope 1) and final slopes (slope 2) indicative of alveolar recruitment and optimal compliance respectively [44], the curves were fitted with R^2^ = 0.9, where R^2^ is the coefficient of determination [45]. Additional local to global behavioral insights were assessed by analyzing intermediate inflation stages of a representative pig at 15BPM and 1350 ml applied volume (Fig. 7A), through topological contour maps (Fig. 7B) and histograms (Fig. 7C).

**Fig. 4:**
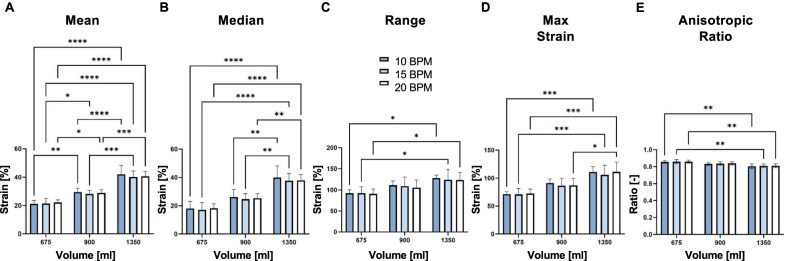
Statistical analysis of mean (**A**), median (**B**), range (**C**), and maximum strains (**D**), and anisotropic ratios (**E**) for varying rates and inflation volumes. Significant volume dependency was observed for all data sets between 675 and 1350 ml, and no significant rate effects were found

Data was analyzed by a two-way repeated measurements ANOVA test (GraphPad Prism) with Bonferroni correction for strain means, medians, ranges, and maximums at various volumes and breathing rates, in addition to anisotropic ratio comparisons (Table 2, Fig. 4). Significance was set at p < 0.05. p-values less than 0.05, 0.01, and 0.001 were represented by *, **, and ***, respectively.

## Results

Specimens’ weights, initial lung volumes, peak lung volumes, and peak pressures for three breathing rates were displayed in Table 1. The mean weight of the lung samples was 903 g. Based on the averages of the four specimens, the amount of air trapped within the lung is 46% of the initial lung volume. Peak pressures experienced for all rates were in the range of 0.36–0.52psi and the peak lung volume inflation due to compressed air ranged 1023.8–1215.3 ml, less than the applied 1350 ml. At the largest applied inflation volume of 1350 ml, the peak pressures increased with increasing breathing rate and alternatively, the lung volume response decreased (10 to 20BPM: peak pressure p = 0.009 and peak volume p  <0.001; 15 to 20BPM: peak pressure p = 0.015 and peak volume p = 0.003).

A representative topological strain colormap was illustrated in Fig. 2. All specimens were observed to experience higher strains at higher volumes as well as greater strains predominantly located where the cranial and caudal lobe meet on the left lung and within the middle lobe of the right lung [46]. Localized high strains often manifested as stripes extending downward towards the lower end of each caudal lobe.

Figure 3 demonstrated the corresponding histograms of the major strain data at maximum inflation; there was a right skewed distribution populating ~ 20% mean strain at lower volumes of 675 ml demonstrating surface homogeneity in comparison to the expanded distribution at increased inflation volume of 1350 ml averaging ~ 40% strain.

Table 2 listed the strain means, medians, maximums, and ranges for each specimen. Statistical significance was found for each rate of inflation as the averaged mean strain values increased with greater inflation volumes (Fig. 4A). Median strain values had statistical significance between the volumes of 675 ml with 1350 ml (10BPM: p < 0.0001, 15BPM: p < 0.0001, 20BPM: p < 0.0001), and 900 ml with 1350 ml (10BPM: p = 0.002, 15BPM: p = 0.003, 20BPM: p = 0.004) for all three rates (Fig. 4B). Average range and maximum strains were also higher with increasing volumes (Fig. 4C and D) and showed statistically significant differences between the lowest and highest inflation volumes of 675 ml and 1350 ml (range—10BPM: p = 0.008, 15BPM: p = 0.029, 20BPM: p = 0.021; strain—10BPM: p < 0.001, 15BPM: p < 0.001, 20BPM: p < 0.001). Breathing rate did not affect the strain means, medians, maximums, and ranges, however those values increased with increasing applied volumes.

**Fig. 5:**
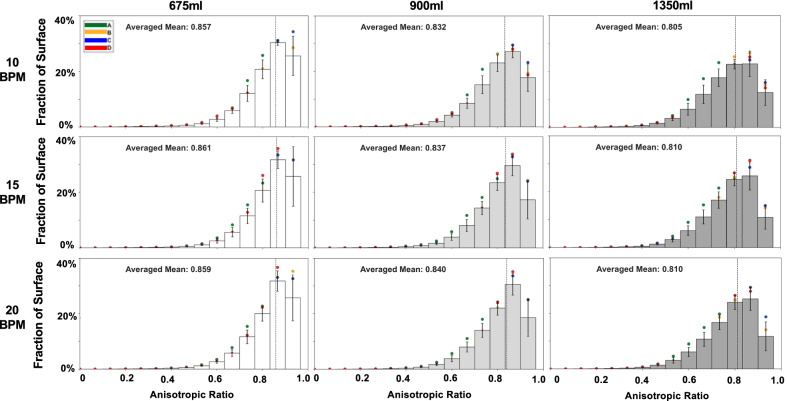
Anisotropic ratio for the fractional surface of lung specimens across varying inflation rates and volumes. An averaged mean ranging 0.81–0.86 was observed. Rate had no effect on the anisotropic ratio however increased inflation volumes decreased the anisotropic ratio (Fig. 4E)

In contrast, the anisotropic ratio decreased for increasing applied volumes, averaging 0.86, 0.84, and 0.81 for 675 ml, 900 ml, and 1350 ml, respectively for 20BPM (Table 2). Breathing rate had a negligible effect on the anisotropic ratio. With increasing volume, the tissue exhibited greater directional expansion preference as defined by the decreasing anisotropic ratio, which was found to be statistically significant between the 675 and 1350 ml volumes (10BPM: p = 0.005, 15BPM: p = 0.007, 20BPM: p = 0.009) (Fig. 4E). The histogram of the anisotropic ratio as a fraction of the lung surface found the lung tissue is near isotropic as the averaged means of the ratios stayed within the range of 0.81–0.86, closer to the value of 1 (Fig. 5). In agreement with previous insights, the specimens primarily expanded in the lateral-medial and ventral-dorsal direction (across the ribcage) [27].

**Fig. 6:**
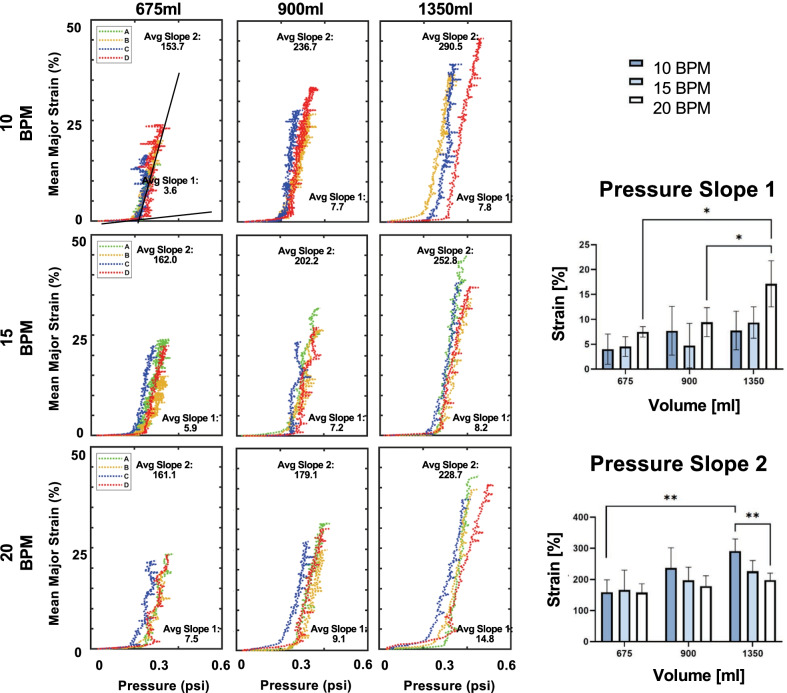
Associated global pressures and local mean strain. The slopes of these bilinear graphs and the calculated inflection points were analyzed for statistical significance to assess local tissue compliancy

Associations between local and global behavior for each specimen was highlighted in Fig. 6. The trend for average slope 1 appears to increase for increasing volumes, however, is only statistically significant for 20BPM when comparing 675 ml and 900 ml to 1350 ml (675 ml: p = 0.011, 900 ml: p = 0.048). Slope 2 also trends higher with increasing volumes however is significant only for volume comparisons of 675 ml and 1350 ml for 10BPM (p = 0.005) and between the rates of 10BPM and 20BPM for 1350 ml inflation volume (p = 0.009). Breathing rates did not generally affect the slopes but decreased slope 2 trends were noted at faster rates for 900 ml and 1350 ml, (significant between 10 and 20BPM for 1350 ml). The mean strain evolution as a function of applied volume plot (not shown) was also analyzed and was found to not be dependent on the rate nor the amount of volume applied.

**Fig. 7:**
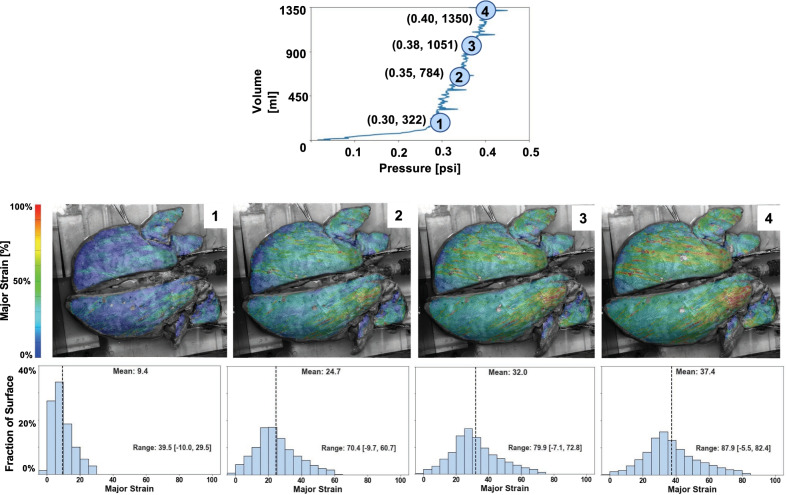
Evolution of the local strain as related to global inflation pressures and volumes. Four intermediate stages of a representative pig lung at 15 BPM inflated to 1350 ml (**A**) was presented as qualitative strain contours (**B**) and quantitative histograms (**C**). Increasing global lung pressure and volumes corresponded to increasing local strain maximum and mean values as well as a larger range of experienced strains. The transition from a right skewed distribution at stage 1 to a more distributed spread of strain values for stages 2–4 was observed and illustrated greater surface heterogeneity

Figure 7A demonstrated the inflation trajectory for an applied volume of 1350 ml of a representative pig at four nominal stages, which linked lung pressures and volumes to the spatial distribution of local strains as exhibited on the contour map (Fig. 7B) and was quantified in the corresponding histograms (Fig. 7C). The evolution of the contour map showed local strains (including the mean, range, and maximums) increased with greater global inflation volume and pressure. The strains were observed to continuously evolve in real-time and resulted in regions of high strain concentration at the middle and upper caudal lobes of the right and left lung respectively. In the select stages corresponding to a global lung inflation volume increase of 322–1350 ml, the global lung pressure rises from 0.30 to 0.40psi, and the maximum strain grows from 29.5 to 79.8%. Histograms illustrated right skewed strain distributions initially with lower mean strain of 9.4% and 0–30% range which transitioned to higher mean strains of 37.4% coupled with increased strain ranges of 0–80% and greater surface heterogeneity.

## Discussion

This work builds upon our previous methods introducing DIC as a novel technique for analyzing topological strains of the whole porcine lung organ and associates quantifications of global pulmonary loading and local strain measures to further characterize porcine surface heterogeneity, anisotropy, and the effect of volume and breathing rate on lung deformations for the first time. Strains as high as 111% are reported with an average of 40% at the greatest inflation tidal volume of 1350 ml. We observe isotropic-leaning organ distension tendencies, and significant local strain dependencies on the global pressures and volumes imposed, especially at the greatest volumes and breathing rates; on the other hand, we observe frequency dependent trends only for a single comparison of local compliancy. Interestingly, we find breathing rates play no significant role in local lung strain values. Findings are specifically translatable to clinical research investigating the role of pulmonary frequency dependencies, such as multi-oscillatory ventilation and inspiratory rate induced lung injury [24, 47].

### Lung surface heterogeneity

Upper regions of the caudal lobe in the left, and middle lobe in the right lung experience higher strains as seen in Fig. 2 [46]. This difference in behavior at the upper and lower regions of the lung have been previously seen in the literature where inflation tests on dog and mice lung found similar regional behaviors of nonuniform air distribution; greater distension is seen in the upper airways of mice where the large bronchi of the left and right lung reside [48, 49]. This regional behavior may be attributable to the bronchi network, which is heterogenous in geometry and material properties [50]; passageways narrow in diameter distally and therefore, air expansion when approaching the distal regions [51]. Similarly, in humans the alveolar size is four times greater in the upper regions compared to the lower regions and can more readily conduct the higher observed lung stretches [52].

Lung surface heterogeneity can also be qualitatively observed in the strain distributions and strain topological maps at various applied volumes and rates (Figs. 2 and 3). The high striped areas of strain can be due to the presence of underlying airways, which alter the deformation behavior of the enveloping parenchymal lung tissue [53]. Post-experimental dissection demonstrated that the left caudal lobe and right middle and upper caudal lobes were heavily populated by large underlying airways corresponding to the striped areas. The greater regional surface strains observed in our experiments may well be caused by this underlying bronchial network, especially as these strain contour hotspots extended distally across the surface as the values increased.

### Volume effects

Our study finds statistically significant evidence of volume dependence, where lung surface strains as well as pressure increases with increasing inflation volume. In a study employing CT scans where porcine lungs were inflated using our same low and high tidal volumes, this increase in local strain in response to increasing applied lung volume was also observed within the parenchyma [54]. Volume-strain interdependence was also seen in cat [55] and mice [56] specimens. This real-time ex-vivo DIC technique additionally offers insights regarding the temporal evolution of strains as the volume is incrementally applied; as such, we observe the spatial expansion of the lower lungs as more air is delivered (Fig. 2). Compared to previous studies finding the highest strain regions in pig specimens to be central-dorsally located [54], we find the greatest concentration of strains to be dorsal but to be more upper than central; this may be attributed to the ex-vivo nature of our experiments compared to lung specimens with an intact chest cavity.

### Rate effects

Our experiments observe that the breathing rate did not significantly affect the local strains (Fig. 4). In comparable DIC experiments conducted on mice specimens, similar rate insignificance was concluded [56]. We do however see significance in rate variations where peak pressure increases and peak lung volume decreases with greater respiratory frequency between the two smaller rates of 10 and 15 BPM compared to 20 BPM (Table 1). This may be attributable to the viscoelastic nature of lung tissue [57], which causes pressure losses at slow breathing rates [55, 58]. The faster rates may not allow the tissue to adequately react and the air to fully permeate throughout the airways, causing higher pressures and lower volumes in the inflated lung. The limited lung studies exploring this phenomenon on isolated tissue strips in tension or compression have found greater stiffness at higher rates [19, 59], which is a similar trend for our whole organ inflation tests: the final slope tends to stiffen with faster breathing rates (not significant, Fig. 6). Despite our study concluding insignificant rate effects, the additional exploration of breathing frequencies is merited as inspiratory rate is one of the main causes of concern in VILI [47].

### Strain anisotropy

Lung surface strain is found to be anisotropic with a ratio ranging 0.81–0.86. Previous studies in the literature consisting of parenchymal tissue samples [60, 61] have concluded lung isotropy. This can partly be due to the presence of isotropically expanding alveoli [62], however, our observed anisotropy, in agreement with our previous conclusions finding reduced expansion of the lung in the cranial-caudal direction, may be attributed to the underlying airway network previously demonstrated to be twice as stiff along the bronchial length compared to circumferentially [15, 27]. While past experiments inflating whole dog lungs filled with saline [63] have concluded isotropy, our air-filled lungs are known to exhibit alternate mechanics not comparable to liquid inflation [64, 65]. Historical studies on air-filled orthogonally tracked markers on rabbit lungs found near equal directional deformations but were technologically limiting and may not capture pulmonary anisotropy at the resolution presented here [66]. A comparison of both air-filled and saline-filled lung tissue observed higher degrees of anisotropy in air, in agreement with our experiments [53].

### Global to local

Utilizing our system, we are able to simultaneously analyze the evolutionary relationship between the local and global lung response of the lungs. Associated mean strains and pressures in Fig. 6 shows how the tissue exhibits a bilinear behavior, similar to existing lung studies on whole mice lungs [27, 56]. This bilinearity is thought to be an effect of alveoli opening and recruitment at low pressures and expansion at high pressures [62], and could also be attributed to the bilinear response observed in pulmonary constituents (i.e. airways, parenchyma, visceral pleura) [14, 67–70].

Our study facilitates insights regarding the spatial distribution of strains on the lung surface at varying pressure values throughout inflation stages (Fig. 7B), which cannot be observed with classical pressure–volume plots. From our histograms (Fig. 7C), we see that low pressures and volumes exhibit predominantly low strains but as global loading increases, a shift occurs where the lung inflates more heterogeneously and strains extend to a wider range of simultaneously existing low and high values. Such insights can support investigations of oscillatory ventilation schemes to better understand concentrated regions of strain and acute respiratory distress syndrome [25].

Ventilator efficacy and pulmonary disease are also dependent on tissue compliancy [25, 71, 72]; high compliance is found in lungs experiencing obstructive diseases such as emphysema and reduced compliance is found in restrictive lung diseases such as fibrosis [73–75]. Previous studies analyzing these global organ compliances from pressure–volume loops have observed lower lung compliance as higher lung volumes were reached due to incremental lung volume gains at higher pressures [74]; however, our local compliance measures linking mean surface strains to global pressures finds that the regional tissue compliance increases at higher inflation volumes as well as at slower breathing rates. This discrepancy is centered on the definition of local tissue versus global organ compliance; the former has not been widely measured but ex-vivo DIC real-time continuous evolutionary measurements between local strains and global loading makes this now possible.

### Limitations

Despite this study being the first of its kind to systematically characterize global and local lung tissue behavior under varied applied volumes and breathing rates in porcine specimens, there are practical limitations relating to replicating physiological loading conditions as seen in past works [76]. First, the lung is explanted and removed from the restraining ribcage. While we anticipate that absolute deformation values may differ from intact thoracic cavity analyses, the relative local strain alterations due to sequential increases in applied loading or faster breathing frequencies is unlikely to be affected. Nonetheless, the ex vivo nature of these experiments must be considered when considering physiological translation, particularly as alveoli surfactants degrade ex vivo and may impact airway recruitment as seen in the inflection points of Fig. 6 [77].

Another limitation in using DIC is that the measurements are topological. Our study lacks the ability to assess internal lung behavior, such as those afforded by digital volume correlation (DVC) or CT scans; however, DVC and CT scans are time intensive and limit real-time observations of continuous strain evolution during a respiratory cycle where qualitative local strain assessments can be regionally observed as instantaneously interfaced with our global pressure volume data, as is the advantage of DIC techniques. Additionally, to maintain the focus of this study, we have limited our analyses to the inflation limb of the respiratory cycle; we note that the deflation portion of lung mechanics may exhibit alternate mechanics and intend to consider such behavior in future works.

## Conclusion

The present study relates real-time topological strain data with global lung organ measurements and finds the local tissue response exhibits volume and pressure loading dependence but rate independence. The first time-continuous measures and associations between the tissue and organ level kinetics and kinematics of the porcine lung provided here inaugurates new insights which can be used in future computational models and subsequent studies seeking to contrast healthy and diseased pulmonary mechanics to improve ventilation techniques.

## Data Availability

The data from this study are available upon reasonable request.

## Abbreviations

COPD: Chronic obstructive pulmonary disease
MV: Mechanical ventilation
VILI: Ventilator-induced lung injuries
DIC: Digital image correlation
DVC: Digital volume correlation
OCT: Optical coherence tomography
CT: Computerized tomography
PV: Pressure–volume
BPM: Breaths per minute
