# Correction to: Examining lung mechanical strains as influenced by breathing volumes and rates using experimental digital image correlation

**DOI:** 10.1186/s12931-022-02050-5

**Published:** 2022-05-23

**Authors:** C. A. Mariano, S. Sattari, K. A. M. Quiros, T. M. Nelson, M. Eskandari

**Affiliations:** 1 Department of Mechanical Engineering, University of California at Riverside, Riverside, CA USA; 2 BREATHE Center, School of Medicine, University of California at Riverside, Riverside, CA USA; 3 Department of Bioengineering, University of California at Riverside, Riverside, CA USA

## Correction to: Mariano et al. Respir Res (2022) 23:92 https://doi.org/10.1186/s12931-022-01999-7

Following publication of the original article [1], the authors identified an error in Fig. 1.

**Fig. 1:**
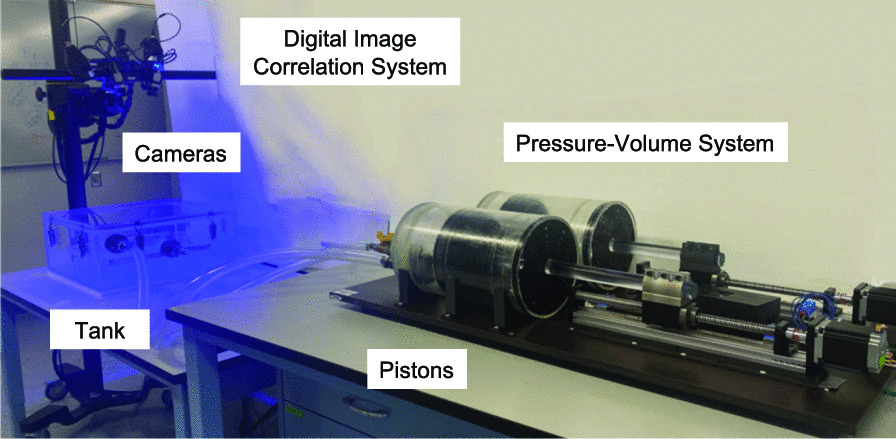
Experimental set-up of the electromechanical pressure–volume ventilation apparatus (right) interfaced with the digital image correlation system (left). The Trilion ARAMIS Adjustable 12M two-camera system is positioned above a transparent, air-tight tank containing the lung specimen which is controlled by the dual-piston apparatus to apply inflation volumes and measure resulting lung volumes and pressures. The combination of these two systems enables the simultaneous collection of global pressures and volumes and local lung topological strain measurements

The word “tan” should be corrected to “tank” in Fig. 1.

The correct version of figure is given.
